# Effect of Transversus abdominis muscle training on pressure-pain threshold in patients with chronic low Back pain

**DOI:** 10.1186/s13102-021-00262-8

**Published:** 2021-04-01

**Authors:** Changming Xu, Zhiwei Fu, Xueqiang Wang

**Affiliations:** 1 Department of Rehabilitation, Renji Hospital Affiliated to Shanghai Jiaotong University School of Medicine, Shanghai, China; 2 Department of Sport Rehabilitation, Shanghai University of Sport, Shanghai, China; 3 Department of Bone and Joint Surgery, Renji Hospital Affiliated to Shanghai Jiaotong University School of Medicine, Shanghai, China; 4 Department of Rehabilitation Medicine, Shanghai Shangti Orthopaedic Hospitai, Shanghai, China

**Keywords:** Transversus abdominis training, Chronic low Back pain, Pressure-pain threshold

## Abstract

**Background:**

Therapeutic training is the most commonly used treatment methods for chronic low back pain (CLBP), and the use of a pressure biofeedback unit for transversus abdominis muscle (TrA) training is one of the core muscle training methods. The study aim of this research is to explore the effects of different intensities (sham training, low-intensity and high-intensity) of TrA muscle training on people with CLBP in pressure-pain threshold (PPT).

**Methods:**

A total of 45 patients with CLBP were recruited, of whom 44 were included in the analysis. Fifteen, 14, and 15 were included in the sham training group, the low-intensity group, and the high-intensity group, respectively. A pressure biofeedback unit was used in performing a one-time TrA training intervention involving 30 times of 180 mmHg TrA contraction training at high intensity for 10 min and 15 times of 100 mmHg TrA contraction training at low intensity for 5 min. The sham training group completed comfort exercises and did not undergo training. The evaluation indicators were as follows: PPT, short-form McGill pain questionnaire, and body surface pain radiation.

**Results:**

High-intensity training could activate more waist core muscles than low-intensity training. Significant changes on PPT (units: kgf) were observed in the following four muscles immediately after high-intensity training: iliopsoas [0.69 (0.13–1.25) 95% CI, *p* = 0.020]; quadratus lumborum [0.84 (0.23–1.45) 95% CI, *p* = 0.012]; erector spinae [0.66 (0.18–1.15) 95% CI, *p* = 0.011]; transversus abdominis [0.70 (0.26–1.14) 95% CI, *p* = 0.004], and in three muscles after low-intensity training: quadratus lumborum [0.61 (0.17–1.05) 95% CI, *p* = 0.009]; transversus abdominis [0.14 (from − 0.15 to 0.43) 95% CI, *p* = 0.022]; piriformis [0.55 (0.13–0.98) 95% CI, *p* = 0.014]. The change in body surface pain radiation immediately after exercise was [− 10.87 (from − 17.51 to − 4.22) 95% CI, *p* = 0.003] for high-intensity training and [− 5.21 (from − 9.40 to − 1.03) 95% CI, *p* = 0.019] for low-intensity training.

**Conclusions:**

TrA training could increase the PPT of the waist core muscles and reduce the radiation range of waist pain. The benefits of high-intensity training are higher than those of low-intensity training.

**Trial registration:**

ChiCTR-TRC-13003701. Registered 18 October 2013.

**Code of ethical approval:** 2018069.

## Introduction

Low back pain (LBP) is one of the most common musculoskeletal disease in clinics, consuming a huge amount of medical resources over the years [1–4]. Nearly everyone has experienced LBP in their lives, and many people who have LBP could recover within 1 year, but some experiences chronic LBP (CLBP) with intermittence or persisting pain of low or medium intensity [1–3]. Thus, the management of symptoms, absenteeism, and decline in daily life activities results in substantial family and social financial losses. Moreover, many patients with CLBP suffer from pain and disability that affect their psychological, social, and physical conditions. These factors alter the pain-processing mechanisms [5–7]. In other words, the central pain-processing mechanisms of some patients who experience pain may become sensitive and exhibit increased neuronal responsiveness to injurious pain mechanism, and this situation results in a disproportionate number of pain complaints [8].

Long-term chronic symptoms could lead to changes in the central processing mechanisms at the neurophysiological level [9, 10]. These changes promote the production of neural plasticity at the spinal cord for adaption to adverse changes, thus affecting psychological, somatosensory, and motor performances. Changes in somatosensory sensation, specifically changes in pressure pain threshold (PPT) and tissue hyperalgesia, occur in patients with CLBP, suggesting that central sensitization mechanisms may be involved [11]. Some researchers believed that persistent nociceptive stimulation is related to cortical and subcortical reorganization and that it plays an important role in the chronic process of LBP [6]. This potential change in somatosensory sensation may be determined by changes in tactile sensitivity caused by changes in the cerebral cortex and neurochemical changes in the process of central sensitization [6, 8–11]. Common dyskinesia and poor treatment response in CLBP may be related to these neurophysiological changes [12].

Therapeutic training is currently the most commonly used treatment methods [13–17], and the use of a pressure biofeedback unit (PBU) for transversus abdominis muscle (TrA) training is one of the core muscle training methods [18–20]. However, some researchers believed that the intensities of activities of people with CLBP are not lower than those of asymptomatic people, and no evidence of direct correlation between the level of physical activity and pain intensity or dysfunction has been found [21]. Whether the effects of different intensities of transverse abdominal muscle training on people with CLBP in PPT is unclear [22]. The current study aimed to explore the effects of different intensities (sham training, low intensity, and high intensity) of transverse abdominal muscle training on people with CLBP in terms of PPT. The results could provide relevant theoretical basis for the clinical treatment of CLBP.

## Materials and methods

**Fig. 1:**
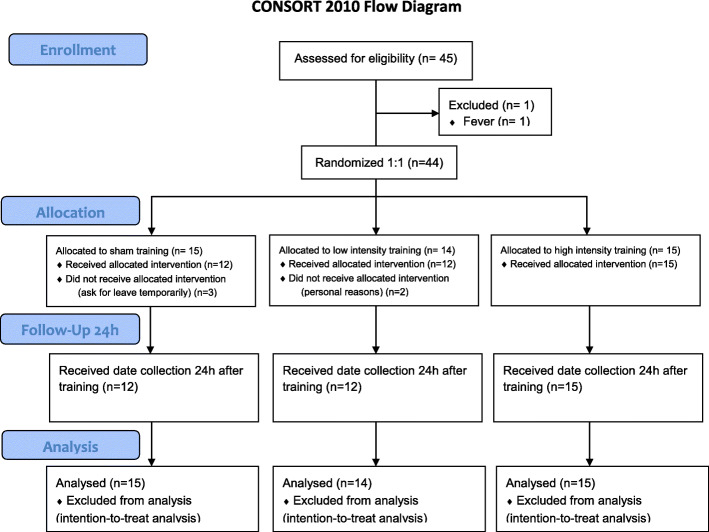
Flowchart of the Training Experiment

Before this study, an experimental plan approved by the Ethics and Research Committee form Shanghai University of Sports (certificate number: 2018069) was formulated. The study was conducted in Shanghai University of Sports and Renji Hospital affiliated to Shanghai Jiaotong University School of Medicine from December 2018 to December 2019. Each subject signed an informed consent prior to entering the study. The specific experimental process is shown in Fig. 1.

### Subjects

Through posters and online recruitment, a total of 45 patients with CLBP were recruited to participate in this experiment. Previous studies about PPT in CLBP were used as references when determining the sample size [23–25]. The effect size provided by Honoré M [21] was chosen (d = 0.56; 95% CI: 00.4–1.08), the α err prod was 0.05, the power (1-β err prod) was 0.80, the number of groups was three, the total sample size was 36, and a 20% loss rate was considered. The final sample size was 45 (calculated by G* Power 3.1.9.2 using F tests), considering that the difference of PPT has at least one standard deviation on average before and after training (95% CIs and 80% predictive power). They were mainly recruited from the Rehabilitation Department of Renji Hospital, which is affiliated to the Shanghai Jiaotong University School of Medicine and Shanghai University of Sport.

The inclusion criteria were as follows: 1) age, 18–50 years; 2) having complaint about CLBP at least 3 months before the study and currently experiencing LBP; 3) visual analogue scale (VAS) score of ≥3 when LBP occurs; 4) Roland Morris Disability Questionnaire score of ≥3; 5) consent to refrain from drinking coffee, tea, milk tea, and other caffeinated drinks during the experiment; 6) no strenuous training a week before the experiment, and no behavior to change training habits; and 7) voluntary informed consent form and ability to complete the experiment. The age range was selected to provide a uniform sample of factors affecting CLBP in a general working population.

The exclusion criteria were as follows: 1) unable to evaluate pain intensity; 2) current use of analgesics, narcotics, or tobacco products; 3) history of previous surgery; 4) neurological diseases; 5) psychological illness or cognitive impairment; 6) pregnancy or preparation for pregnancy; 7) having pain in other parts of the body, including tennis elbow, knee-ankle joint pain, fracture, and cervical spondylosis; 8) other clear causes of LBP, such as spinal canal stenosis, ankylosing spondylitis, intervertebral disc herniation, and sciatica; and 9) cold and fever.

The withdrawal criteria were as follows: 1) could not complete the training program in accordance with the process; 2) could not complete the trial for personal reasons; 3) exercises that break the daily routine, including occasional strenuous exercise; 4) seeing a doctor or taking other treatments for LBP; 5) and worsening of CLBP symptoms, such as increased pain or dysfunction.

### Procedure

After the subjects passed the preliminary screening for recruitment, they were randomly selected. Then, baseline data were collected, mainly including demographic information, physical activity self-report questionnaire, PPT, body surface pain radiation range, and short-form McGill pain questionnaire. A series of transverse abdominal muscle training was performed at different intensities. The rating of perceived exertion (RPE), PPT, body surface pain radiation range, and short-form McGill pain questionnaire scores were collected immediately after training and 24 h after training. Statistical analyses were performed after all data were collected and archived.

All evaluators underwent systematic learning and training, and they were assisted by qualified physical therapists. The evaluators had no prior information about the purpose of the training. Evaluator 1 was responsible for collecting baseline data, evaluator 2 was responsible for training procedures, and evaluator 3 was responsible for collecting the PPT, short-form McGill pain questionnaire, and body surface pain radiation range. They were blinded to the experimental conditions. Each subject underwent separate training, and the data of each subject were collected separately. All subjects were instructed to refrain from discussing anything about the training. They were trained in designated rehabilitation centers.

### Training program

**Fig. 2:**
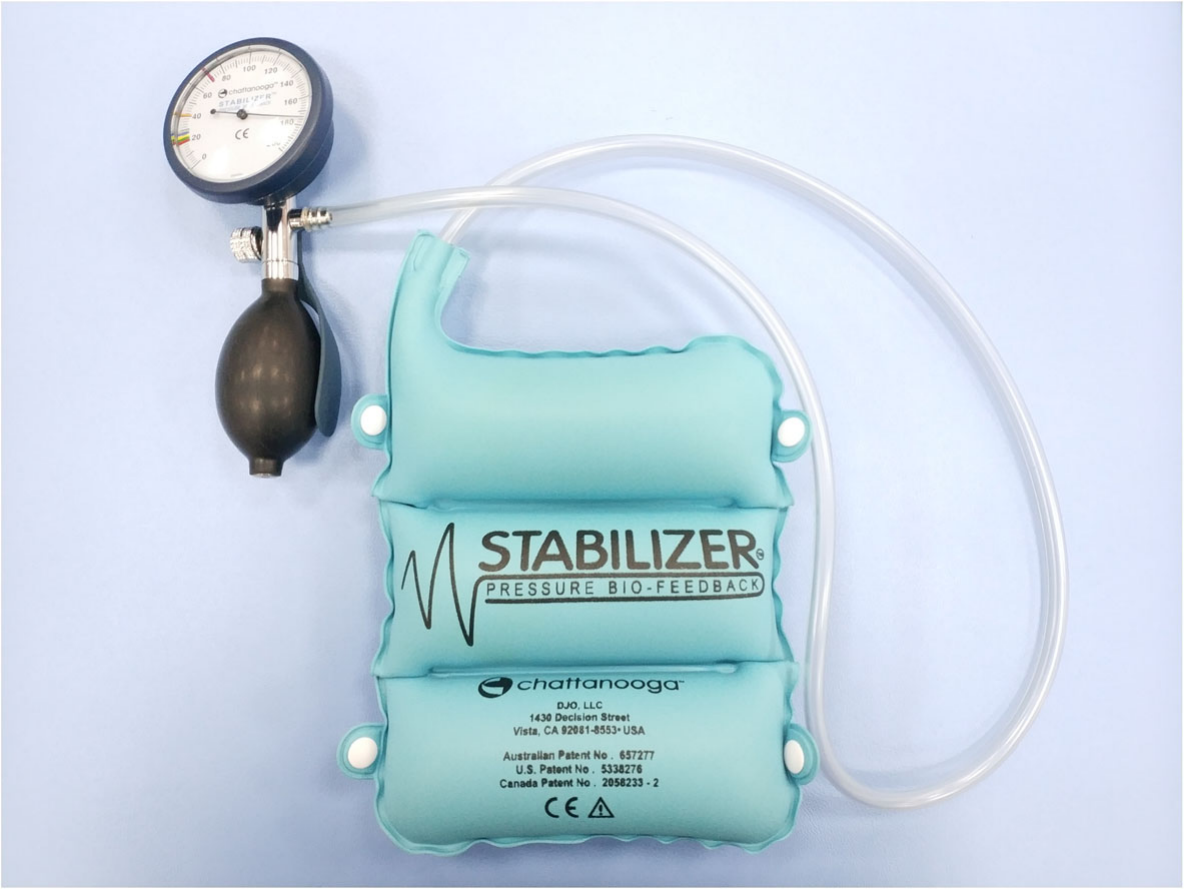
Pressure Biofeedback Unit

PBU (Fig. 2) was used in the training of the transverse abdominal muscle. As a tool for core muscle training, PBU is widely used in the field of CLBP treatment, and it has good reliability and validity [26].

#### Transversus abdominis muscle training

**Fig. 3:**
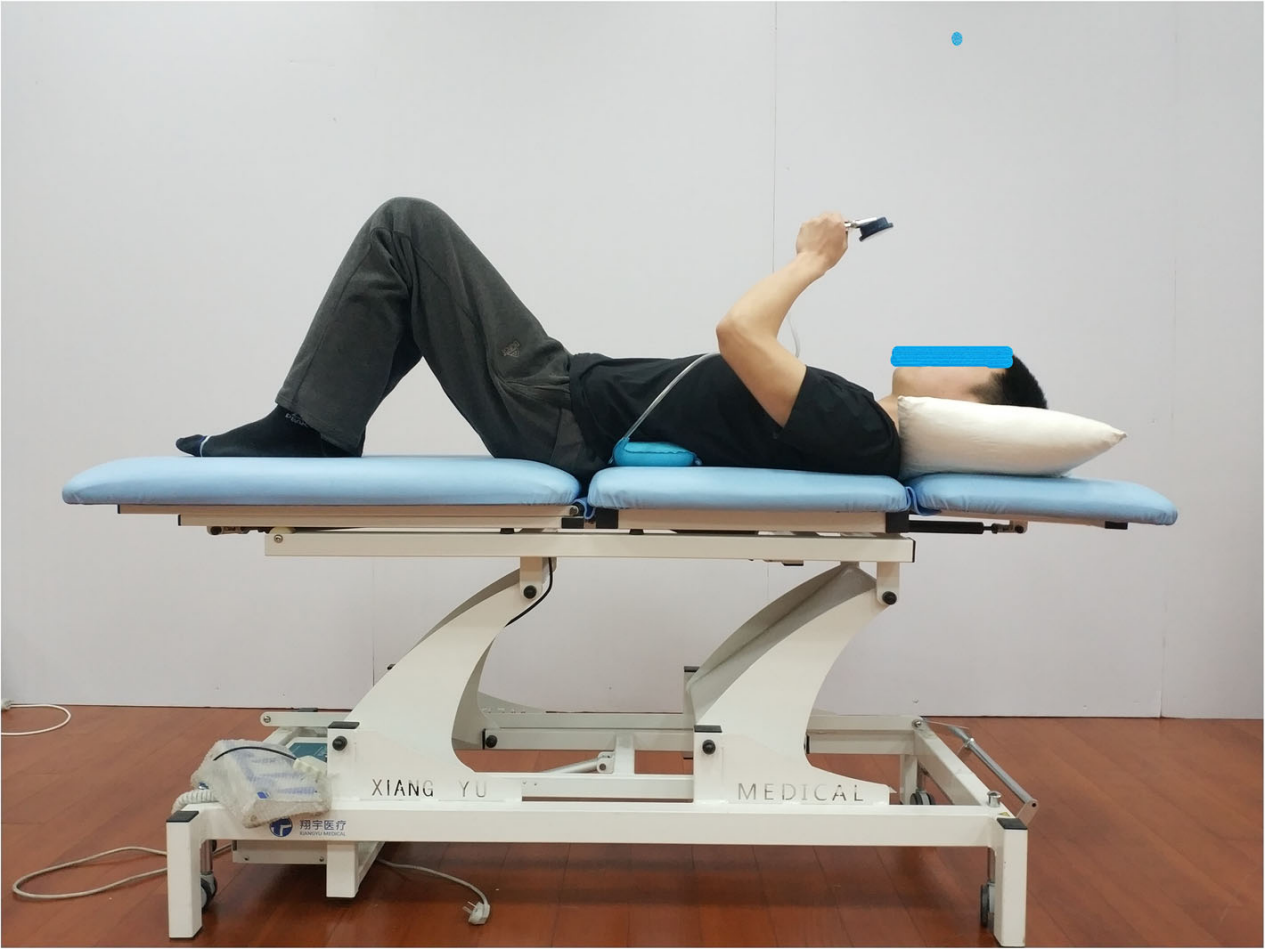
Transversus Abdominis Muscle Training

**Fig. 4:**
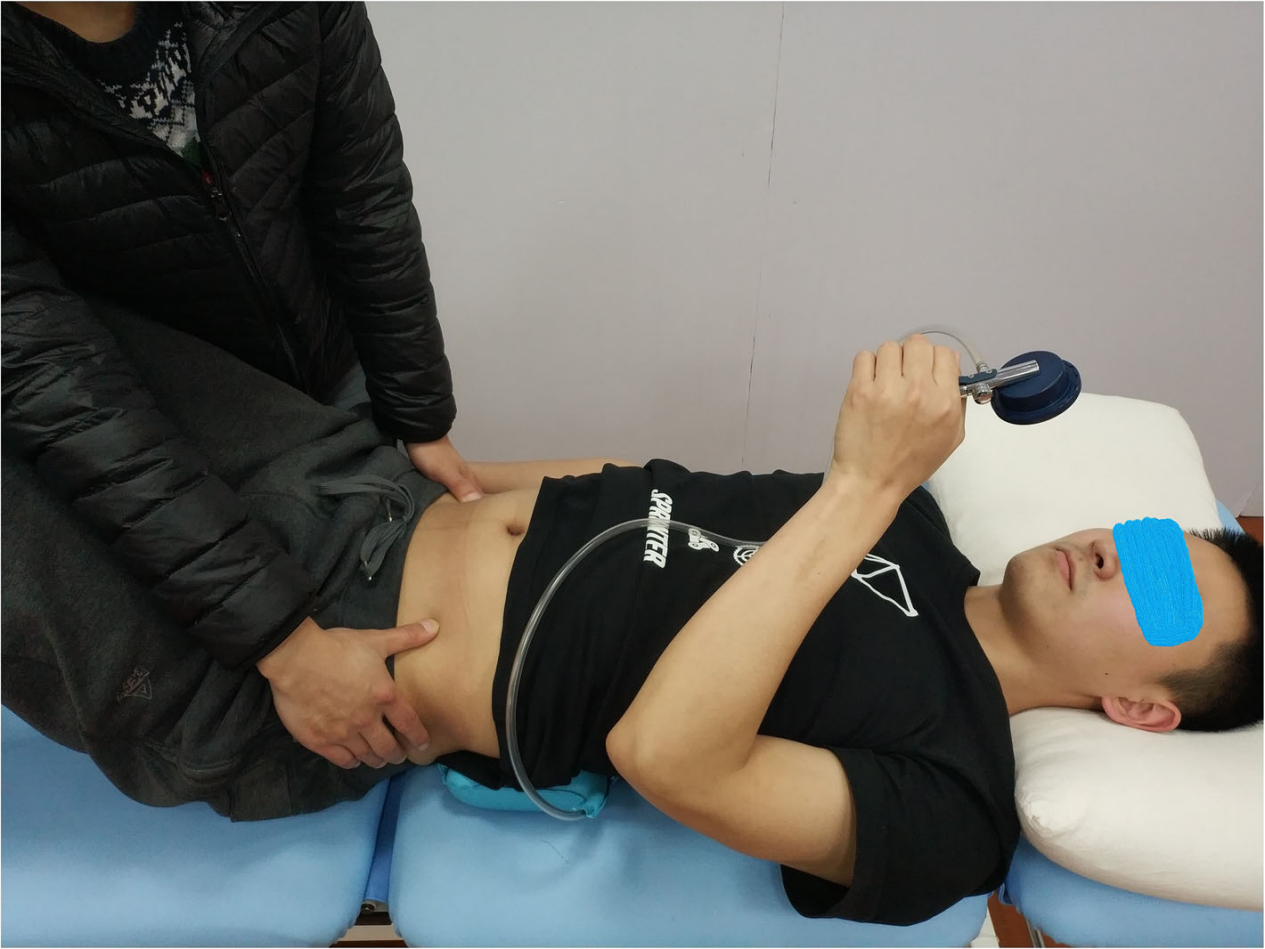
Palpation

The subjects laid on the treatment plane with knees bent, and the nose tip, sternum, and navel were aligned in a straight line. Their feet were flat on the treatment plane. The therapist instructed the subjects to inhale, exhale, and gently retract the navel towards the spine to indent the abdomen. Little or almost no movement of the pelvis was ensured, and the lower ribs were turned or pressed down. The chest was not sucked or lifted, and no increase in foot pressure was ensured, as shown in Fig. 3. The researchers touched the muscle at the intersection of the anterior superior iliac spine’s horizontal line and the lateral rectus abdominis muscle, and the transverse abdominis muscle felt flat tension during contraction [27, 28] (muscle bulging could be felt by contraction of the transverse abdominis muscle), as shown in Fig. 4. The researchers provided the following instructions to the subjects: tighten navel stick to back, use back to press the air bag, let pointer reach a specified value, stabilize, tighten the buttocks, lift the anus, and prevent the pointer from floating. The researchers reminded the subjects to keep their spine stable during the transverse abdominis muscle training.

Before the training, the PBU was inflated to 40 mmHg. The experimenter completed air tightness check to ensure that the PBU was not leaking.

Before the formal training, the subjects were instructed to familiarize themselves with the training until they were able to complete the contraction training of the transverse abdominis. The entire training process was completed under the full guidance of the researchers for the subjects to be able to complete the training accurately and effectively.

#### Different intensity training programs

High-intensity group: the PBU pointer reached 180 mmHg or the subjects tried their best. The movements were performed for 15 s each, and the subjects rested for 5 s between the movements. The movement was performed 30 times, with a total of 10 min.

Low-intensity group: the PBU pointer reached 100 mmHg, and 15 movements were performed for 15 s each. A 5-s rest was performed between the movements, and the total time was 5 min.

Sham training group: the subjects were instructed to lie on the treatment plane, as indicated in Fig. 3. All the procedures were the same as those in the high- and low-intensity groups, except that no contraction of the transverse abdominis muscle was performed. The researchers instructed the subjects to lie on the treatment plane without other movements to prevent the pointer from floating.

No difference was found between men and women in the different intensity training groups. A difference of 80 mmHg between the high- and low-intensity groups was ensured to distinguish the training intensity effectively. Additional 15 movements were completed, and the training time was more than 5 min. After the training was completed, each subject filled the RPE, which could effectively be used in distinguishing training intensity [29].

### Outcome measures

The baseline data mainly collected were age, sex, height, weight, years of education, sedentary, regular training, duration of each training (minutes), weekly activity frequency, and self-perceived training intensity (0–6: 0, rest; 1, very weak; 2, mild; 3, medium; 4, fatigue; and 5, very tired).

**Fig. 5:**
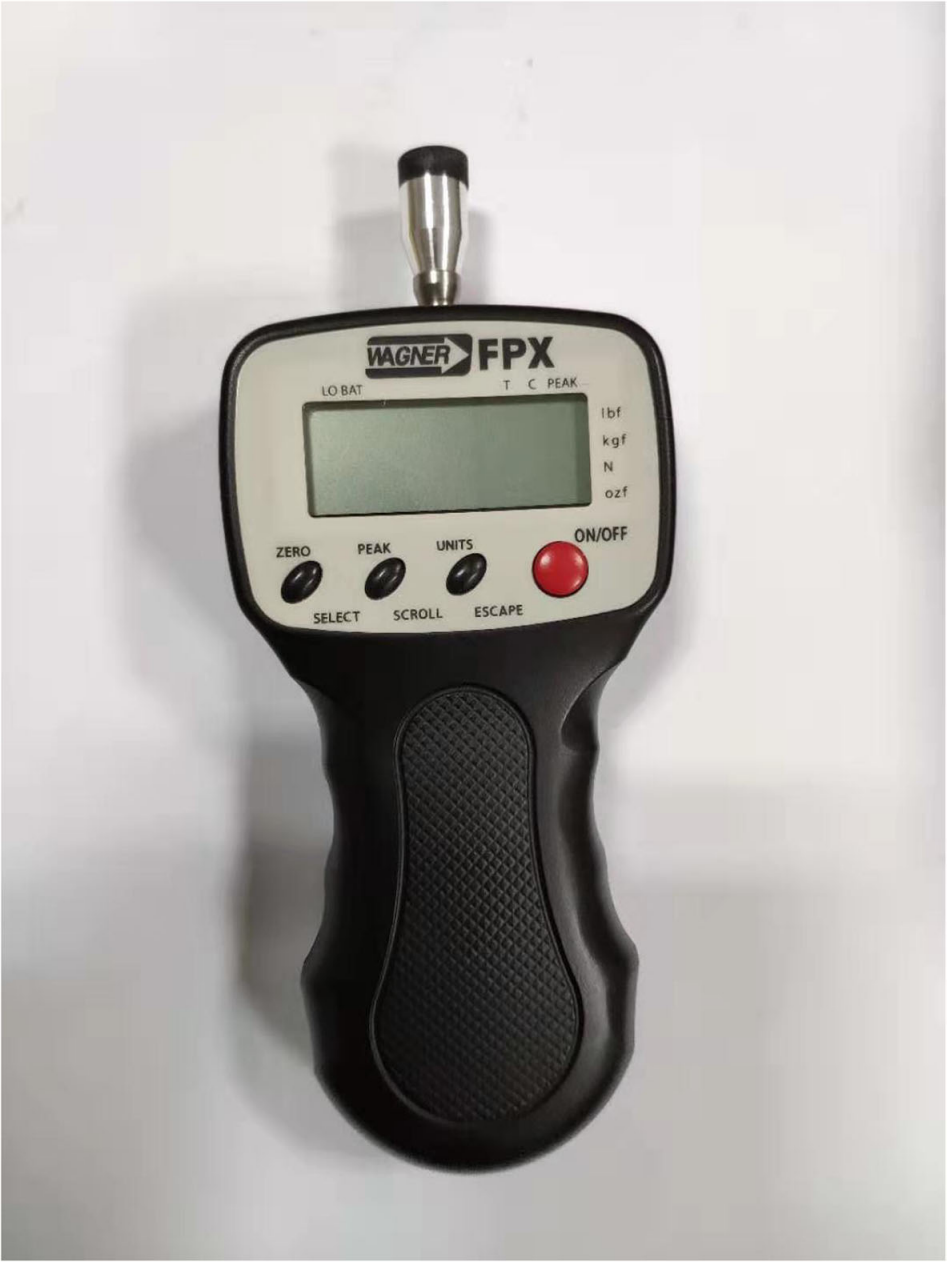
Pressure-pain Threshold Texter

**Fig. 6:**
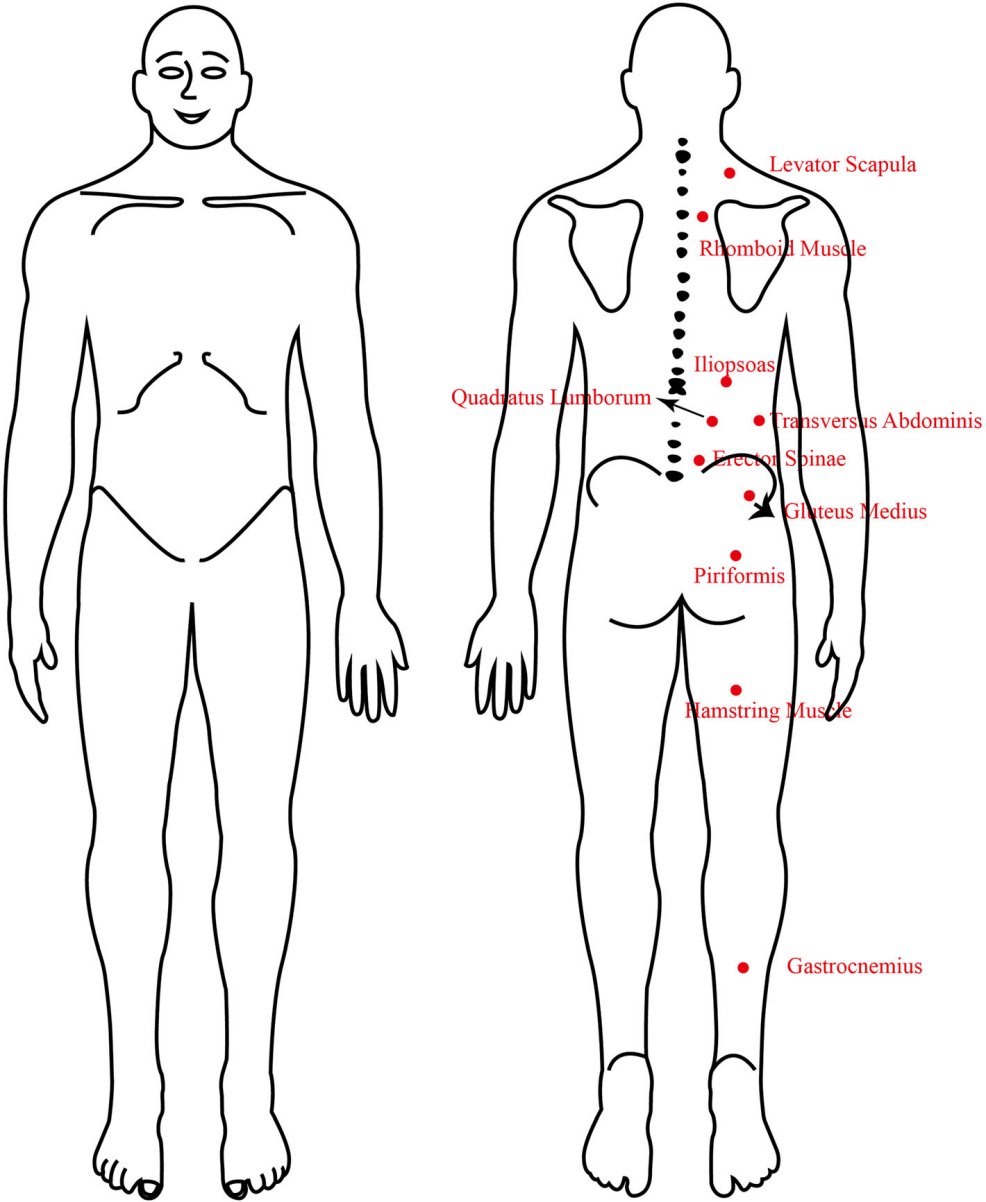
Diagram of Muscle Test Points

First, baseline data were collected from the subjects, followed by PPT data by using a handheld pressure pain tester (FDX 25 FORCE GAGE ​​25 × 0.02 Ibf, Fig. 5) with a 1 cm^2^ replaceable rubber flat probe. The reliability of PPT measurement was acceptable [30], its intra- and intersessions were perfect with ICC (0.85–0.99) [31, 32]. PPT was used to stimulate the corresponding muscles at a constant speed. Stimulation was immediately stopped when the subjects felt pain. Each measurement was repeated four times, and the average value was obtained. After the subjects were familiarized with the test at their dominant arm, PPT was collected from the painful side. It was collected from the dominant side when the pain was in the middle area or symmetrical on both sides. As shown in Fig. 6, data from the following muscles were collected: levator scapula (two fingers above the upper scapula), rhomboid muscles (the midpoint of the connection between the scapular spine and third thoracic spinous process), iliopsoas muscle (the psoas major muscle next to the transverse process of the first lumbar vertebra), quadratus lumborum muscle (near the thick part of the iliopsoas muscle of the third lumbar spinous process), erector spinae (horizontal fifth lumbar vertebrae paraspinous), transversus abdominis (the intersection of the vertical line of the iliospinale posterior height and the horizontal line of the transverse process of the third lumbar vertebra), gluteus medius (the muscle depression below the posterior superior iliac spine), piriformis (The depression of the ischial foramen), hamstring muscle (the four fingers below the midpoint of the gluteal line), and gastrocnemius muscle (the place where the calf muscle is rich).

Data from the iliopsoas muscle, psoas muscle, vertical spine muscle, transverse abdominis muscle, gluteus medius muscle, and piriformis muscle were obtained because these muscles are common pain areas in patients with CLBP, and they are important core muscles for stable posture [33–35]. The levator scapulae and rhomboid muscles are proximal muscle reference points, whereas the hamstring and gastrocnemius muscles are distal muscle reference points. Both points were used in determining whether transverse abdominal muscle training has a central inhibitory and analgesic effect on CLBP.

**Fig. 7:**
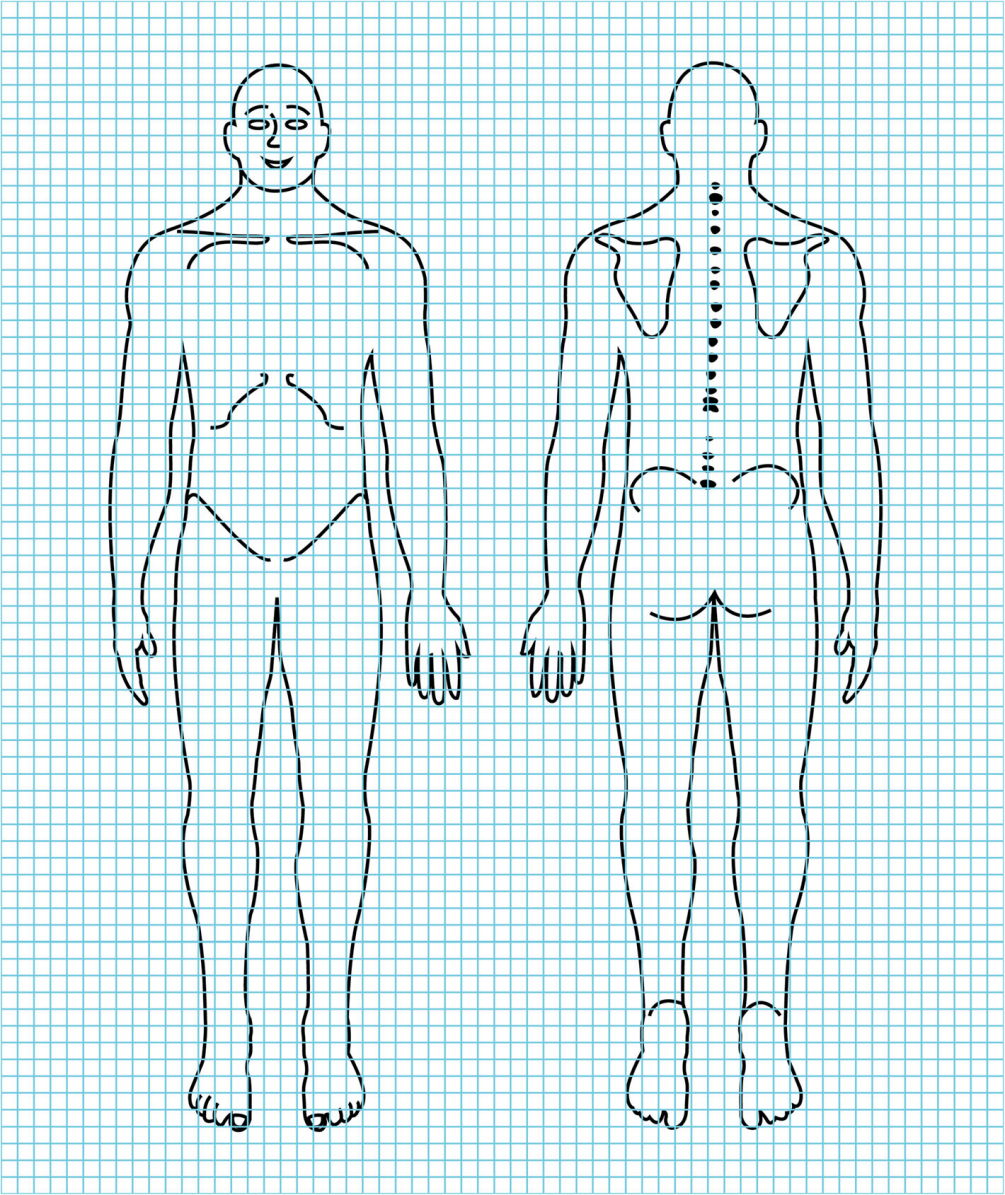
Figure of Body Surface Pain Radiation

Pain tolerance measurement involved 120% PPT continuous stimulation at the erector spinae measurement point for 1 minute [36]. During the test, the subjects were asked to pay attention to the nature of the pain, pain range, and pain level. Then, they were asked to answer the figure of body surface pain radiation [37] (Fig. 7) and the short-form McGill pain questionnaire [36] immediately. The subjects were asked to circle the pain range on the figure of body surface pain radiation, and the data were evaluated in accordance with the number of grids circled by the subject. After the test was completed, the subjects were instructed not to train vigorously and to refrain from drinking caffeinated beverages, such as coffee and tea. The test was repeated after 24 h.

### Statistical analysis

Microsoft Excel 2019 was used in recording preliminary data, and IBM SPSS Statistics 20.0 was used for detailed statistical analysis after verification and classification.

Before statistical analysis was conducted, the Shapiro–Wilk test was used for initial testing of the data for normal distribution, and the data that did not conform to normal distribution were deleted. One-way ANOVA was performed to select the mean difference (post-intervention minus pre-intervention), and if no difference was found, no post-hoc analysis was performed. If a significant difference was found, a post-hoc analysis was performed. LSD was selected for post-hoc analysis. The results were expressed as mean ± standard deviation (M ± SD), and the corresponding F and *p* values were marked. A *p* value < 0.05 was considered significant. Multiple paired t-tests were used in analyzing differences within each group (pre, immediate, 24 h). The correction family-wise error rate was adjusted to a *p* value such as 0.05/2 of comparisons. The results were expressed as mean value and 95% confidence interval, and the corresponding *p* value was marked.

Intention-to-treat analysis was used in subjects who completed baseline data collection but did not complete all tests or withdrew from the study. All subjects who completed randomization were included in the analysis, and the last data were used for statistical analysis.

## Results

A total of 45 subjects were included in this study. One subject failed to complete the test performed on the following day because of fever, whereas the remaining 44 subjects completed all the experiments. A normal distribution test was performed before the data were analyzed. Three subjects in the sham training group did not receive allocated intervention (asked for temporary leave), and two subjects did not complete the training (withdrew for personal reasons). A total of 39 subjects received allocated intervention, 12, 12, and 15 of whom were in the sham training group, the low-intensity group, and the high-intensity group, respectively.

### Baseline data

No significant difference in baseline data was observed among the three groups. Age group and gender were evenly distributed, and no significant differences in height, weight, and education years were observed. Likewise, no significant difference in daily activity training information (sedentary, regular training, duration of each training, activity frequency, and self-perception intensity) was observed among the subjects. The detailed statistics are shown in Table 1. No significant differences were found in the baseline data of PPT and the short-form McGill pain questionnaire among the three groups, as detailed in Table 2.

**Table 1:** Baseline Data. Values are (M ± SD) unless stated otherwise

	Sham Training (*n* = 15)	Low Intensity (*n* = 14)	High Intensity (*n* = 15)	F	*p*
Age/years	22.53 ± 2..03	22.36 ± 1.91	22.73 ± 2.79	0.099	0.906
Male/n (%)	8 (53.33)	8 (57.14)	7 (46.67)	0.154	0.857
Female/n (%)	7 (46.67)	6 (42.86)	8 (53.33)		
Height/cm	170.93 ± 6.02	171.14 ± 11.07	169.27 ± 9.38	0.192	0.826
Weight/kg	62.26 ± 11.33	65.00 ± 12.62	59.60 ± 13.26	0.684	0.510
Education/years^a^	4.73 ± 1.53	4.57 ± 1.74	4.40 ± 1.84	0.142	0.868
Sedentary/n (%)	10 (66.67)	9 (64.28)	8 (53.33)	0.301	0.742
Regular training/n (%)	10 (66.67)	9 (64.28)	10 (66.67)	0.011	0.989
Duration of each training/mins	52.33 ± 34.37	65.71 ± 36.52	58.00 ± 34.93	0.524	0.595
Frequency/times per week	3.33 ± 1.79	3.64 ± 1.82	3.00 ± 1.65	0.486	0.619
Self-perceived intensity ^b^	2.60 ± 1.18	3.21 ± 1.05	2.73 ± 1.10	1.207	0.310

^a^: Freshman was 1, sophomore was 2, junior year was 3, senior year was 4, first year of postgraduate was 5, and so on

^b^:0–6 points; 0, rest; 1, very weak; 2, mild; 3, medium; 4, tiredness; 5 very tired

**Table 2:** Baseline Data of Pressure-Pain Threshold and Short-form McGill Pain Questionnaire. M ± SD Units: kgf.

	Sham Training (*n* = 15)	Low Intensity (*n* = 14)	High Intensity (*n* = 15)	F	*p*
Levator scapula	3.92 ± 1.53	3.96 ± 1.65	4.03 ± 1.57	0.018	0.982
Rhomboid muscle	4.64 ± 1.72	4.86 ± 1.89	4.52 ± 1.67	0.141	0.869
Iliopsoas	5.29 ± 2.00	5.67 ± 2.08	4.95 ± 1.92	0.464	0.632
Quadratus lumborum	5.76 ± 2.07	6.43 ± 2.06	6.15 ± 2.19	0.379	0.687
Erector spinae	7.32 ± 2.85	7.60 ± 2.40	7.96 ± 2.12	0.252	0.778
Transversus abdominis	5.75 ± 2.00	5.82 ± 2.14	5.60 ± 2.24	0.041	0.960
Gluteus medius	6.47 ± 2.01	6.14 ± 1.65	5.99 ± 1.55	0.294	0.747
Piriformis	6.73 ± 2.01	6.80 ± 1.67	6.26 ± 1.90	0.314	0.732
Hamstring muscle	7.12 ± 2.69	7.00 ± 2.10	5.85 ± 2.25	1.296	0.285
Gastrocnemius	5.28 ± 1.56	5.78 ± 1.29	4.96 ± 1.48	1.181	0.317
Body surface pain radiation	6.80 ± 8.22	18.57 ± 21.02	17.00 ± 11.13	2.932	0.065
Short-form McGill Pain Questionnaire^a^					
Sensory items	4.87 ± 2.16	5.45 ± 3.01	5.80 ± 3.75	1.122	0.579
Emotion items	1.27 ± 1.22	2.29 ± 2.89	1.20 ± 1.57	1.324	0.277
VAS^b^	4.63 ± 2.78	5.46 ± 2.00	4.20 ± 2.21	1.065	0.354
Present pain intensity^c^	0.73 ± 0.70	0.71 ± 0.61	1.00 ± 0.85	0.710	0.497

^a^:Assessed pain during 120% PPT stimulation; ^b^: Pain score at 120% PPT stimulation for 60 s; ^c^: Pain score at the erector spinae stimulation point 1 min after the pain tolerance threshold was measured

### Effects of different training intensities

**Fig. 8:**
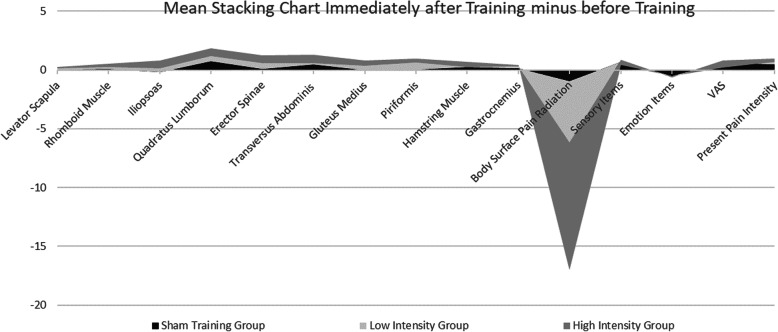
Mean Stacking Chart Immediately after Training minus before Training

The PPT of each group of muscles increased after the training, and the highest increase was observed in the high-intensity group. Significant improvement was noted in the area of pain radiation, and the highest degree of improvement was observed in the high-intensity group. The score in short-form McGill pain questionnaire’s sensory item slightly increased, whereas that in emotion items decreased. Pain of the same intensity stimulated a slight increase in VAS. Other researchers notably reported similar findings, that is, patients with CLBP are prone to fatigue in the waist muscles and increase in such may be due to the sharpness of local muscle fatigue after the training task [38]. Details were shown in the stacking chart immediately after the training versus before training of the mean value at different training intensities (Fig. 8).

Immediately after the training, significant differences in the PPT of the TrA muscle (F = 3.489, *p* = 0.041), the body surface pain radiation (F = 5.512, *p* = 0.008), the present pain intensity (F = 8.047, *p* = 0.001), and VAS (F = 3.534, *p* = 0.038) were noted between groups. Among them, a significant difference was found between the sham training group and high-intensity group in terms of PPT of the TrA muscles [− 0.56 (from − 1.06 to − 0.05), *p* = 0.046], body surface pain radiation [9.93 (3.87–15.99), *p* = 0.002], present pain intensity [0.73 (0.30–1.17), *p* = 0.001] and VAS [1.23 (0.29–2.18), *p* = 0.012]. A significant difference between the low- and high-intensity groups was found in the present pain intensity [0.77 (0.33–1.21), *p* = 0.001]. Further details are shown in Table 3.

**Table 3:** Items with Significant Differences in Pressure-Pain Threshold before and Immediately after Training between Each Group^a^

Variable	Sham training	Low intensity	High intensity	F	***p***	Mean between-group difference		
	***n*** = 15	***n*** = 14	***n*** = 15			Sham training vs. low intensity	Sham training vs. high intensity	Low intensity vs. high intensity
**Transversus abdominis**								
Baseline mean (SD)	5.75 (2.00)	5.82(2.14)	5.60 (2.24)	–	–	–	–	–
Mean score (SD)	6.20 (1.85)	5.97 (2.07)	6.30 (2.01)	–	–	–	–	–
Mean change from baseline (95%CI)	0.45 (0.70 to 0.83)	0.14 (−0.15 to 0.43)	0.70 (0.25 to 1.14)	3.489	0.041	0.31 (−0.19 to 8.17)	−0.56 (−1.06 to − 0.05)^b^	−0.24 (− 0.74 to 0.25)
**Body surface pain radiation**								
Baseline mean (SD)	6.80 (8.22)	18.57 (21.02)	17.00 (11.13)	–	–	–	–	–
Mean score (SD)	5.87 (7.18)	13.36 (15.66)	6.13 (4.61)	–	–	–	–	–
Mean change from baseline (95%CI)	−0.93 (−2.16 to 0.30)	−5.21 (−9.40 to −1.03)	− 10.87 (− 17.51 to −4.22)	5.512	0.008	4.28 (− 1.89 to 10.45)	9.93 (3.87 to 15.99)^b^	5.65 (− 0.52 to 11.82)
**Present pain intensity**								
Baseline mean (SD)	0.73 (0.70)	0.71 (0.61)	1.00 (0.85)	–	–	–	–	–
Mean score (SD)	1.20 (0.78)	1.21 (0.98)	0.73 (0.59)	–	–	–	–	–
Mean change from baseline (95%CI)	0.47 (0.18 to 0.75)	0.50 (0.06 to 0.94)	−0.27 (−0.52 to − 0.01)	8.047	0.001	−0.03 (− 0.47 to 0.41)	0.73 (0.30 to 1.17)^b^	0.77 (0.33 to 1.21)^b^
**Visual analogue scale**								
Baseline mean (SD)	4.63 (2.78)	5.46 (2.00)	4.20 (2.21)	–	–	–	–	–
Mean score (SD)	5.20 (2.48)	5.64 (1.64)	3.67 (1.88)	–	–	–	–	–
Mean change from baseline (95%CI)	0.57 (−0.19 to 1.33)	0.18 (−0.46 to 0.82)	− 0.53 (−1.34 to 0.27)	3.534	0.038	0.46 (− 0.51 to 1.42)	1.23 (0.29 to 2.18)^b^	0.78 (− 0.19 to 1.74)

^a^ Data analysis was performed by 1-way ANOVA, select LSD for post-hoc analysis

^b^ The significance level of the mean difference is 0.05

No significant change was found in the sham training group. In the low-intensity group, the difference between immediately after training and before training was significant in the following: quadratus lumborum [0.61 (0.17–1.05), *p* = 0.009], transverse abdominis [0.14 (from − 0.15 to 0.43), *p* = 0.022], and piriformis [0.55 (0.13–0.98), *p* = 0.014]. In the high-intensity group, the difference between immediately after training and before training was significant in the following: iliopsoas muscle [0.69 (0.13–1.25), *p* = 0.020], quadratus lumborum [0.84 (0.23–1.45), *p* = 0.012], erector spinae [0.66 (0.18–1.15), *p* = 0.011], and transversus abdominis [0.70 (0.26–1.14), *p* = 0.004]. Exactly 24 h after the training, only the high-intensity group showed a significant difference in the quadratus lumborum [0.65 (0.14–1.17), *p* = 0.018]. In terms of the body surface pain radiation range, both groups showed significant decreases as follows: low-intensity training immediately after training [− 5.21 (from − 9.40 to − 1.03), *p* = 0.019] and 24 h after the training [− 7.29 (from − 13.31 to − 1.27), *p* = 0.021], high-intensity training immediately after the training [− 10.87 (from − 17.51 to − 4.22), *p* = 0.003], and 24 h after the training [− 9.87 (from − 16.43 to − 3.31), *p* = 0.006]. The improvement in the high-intensity group was higher than that in the low-intensity group. Further details are shown in Table 4.

**Table 4:** Items with Significant Differences in Pressure-pain Threshold before and after Training in Each Group*. Units: kgf

		Immediately after training		24 h after training	
Group	Item	^**−**^X (95%CI)	*p*	^−^X (95%CI)	*P*
Sham training	–	–	–	–	–
Low intensity	Quadratus lumborum	0.61 (0.17–1.05)	0.009	–	–
	Transversus abdominis	0.14 (−0.15–0.43)	0.022	–	–
	Piriformis	0.55 (0.13–0.98)	0.014	–	–
	Body surface pain radiation^a^	−5.21 (from −9.40 to −1.03)	0.019	−7.29 (− 13.31 to − 1.27)	0.021
High intensity	Iliopsoas	0.69 (0.13–1.25)	0.020	–	–
	Quadratus lumborum	0.84 (0.23–1.45)	0.012	0.65 (0.14–1.17)	0.018
	Erector spinae	0.66 (0.18–1.15)	0.011	–	–
	Transversus abdominis	0.70 (0.26–1.14)	0.004	–	–
	Body surface pain radiation^a^	−10.87 (from −17.51 to −4.22)	0.003	−9.87 (from −16.43 to −3.31)	0.006

*: The correction family-wise error rate was done by adjusted *p*-value 0.05/2. “-” Data with no significant difference is not displayed in the Table 4

^a^: Items of the Short-form McGill Pain Questionnaire

## Discussion

CLBP coexists with pain for a long time, and this state makes the perception of pain between patients with CLBP and normal people different [39, 40]. The present study analyzed in detail the effects of TrA training on PPT and pain tolerance domain in patients with CLBP.

High-intensity training could significantly improve the body surface pain radiation of patients with CLBP and increase muscle PPT. Significant changes were found in the intra-group comparison of pain changes before and after training at different exercise intensities. The high-intensity group had more significant differences than the low-intensity group, while the sham training group had no significant difference. Figure 8 shows a slight change in the sham training group, indicating a certain placebo effect. The muscles that changed in the group were mainly iliopsoas, quadratus lumborum, erector spinae, and TrA, indicating that the use of PBU to train TrA was effective in training the corresponding core muscle groups. Body surface pain radiation showed a significant reduction. After the training, the average values of the subjects’ present pain intensity, VAS, and sensory items scores increased slightly, indicating that the number of neurons that could be recruited from the waist increased after TrA training and PPT could be stimulated accurately. Significant differences were found between the sham training group and the high-intensity group in terms of TrA’s PPT, body surface pain radiation area, present pain intensity, and VAS. The above data showed that TrA training is an reliable core muscle training method that could simply and effectively stimulate related core muscles.

In the experimental preparation phase, some subjects may compensate when they perform high-intensity training compared with low-intensity training. Therefore, during the familiarization phase of the subject, the experimenter should ensure that the subject mastered the transverse abdominis training without compensation. When performing high-intensity training, the experimenter should accurately palpate, remind the subject to keep the spine stable, and ensure that the transverse abdominis could be accurately trained. No subject reported muscle soreness in this experiment, and this situation requires attention in the next experiment.

The stability and coordination of the lumbar core muscles is an important condition for maintaining the stability of the lumbar spine. TrA training could effectively activate the core muscles, correct poor postures and maintain the stability of the lumbar spine, thus improving the PPT of patients with CLBP [20, 41] Grooms DR [20] used PBU to train the transverse abdominal muscles of patients with LBP and pointed out that this training method has good sensitivity and moderate specificity. França FR [41] revealed that training of the transverse abdominal muscles could effectively relieve pain in patients with LBP, alleviate disability, and improve quality of life. Park KN [42] studied the use of PBU in TrA training during active prone knee flexion and found that TrA training could effectively adjust the strength of the waist and abdomen core muscles, thereby improving their coordination and stability, enhancing the control and correction of the pelvis position, and reducing waist pain. Von Garnier K [43] used PBU to measure the recruitment test of transverse abdominis muscles and mentioned that during deep core muscle training, the other core muscles of the waist and abdomen have obvious coordinated contraction. In a study of P Hodges [28] and C Richardson [27], subjects were asked to contract the pelvic floor muscles and gluteal muscles by tightening the levator anus and buttocks during the transverse abdominis training. The results showed improved training effects. Cynn HS [44] used PBU for lumbar stability training and detected the surface electromyography signals of the quadratus lumborum, gluteus medius, TrA, external oblique, rectus abdominis, and multifidus muscle contractions. The present study obtained similar results. After TrA training, the tenderness threshold of other the waist core muscle groups changed because the muscles worked together. The pain areas of the reference muscles, namely, the levator scapula, rhomboid, hamstring, and gastrocnemius muscles, showed no notable changes, and no central nervous system inhibition of pain was observed. The possible reason is the low intensity and duration of the task. Changes in areas farther from the training area occurred after excessive or strenuous exercise [38]. This finding suggested that training intensity could be further increase for good results.

A study comparing subjects with acute and chronic LBP and with PPT [45] showed that those with CLBP had extensive pressure hyperalgesia in the waist area, whereas the those with acute LBP did not have this condition. Similarly, a similar phenomenon was noted in the present study. Patients with CLBP were more prone to generalized radiation and had longer residual time than those with acute LBP. This phenomenon may have been caused by long-term pain experienced by patients with CLBP; this condition indicated that the central nervous and peripheral sensory systems underwent adaptive changes [45, 46]. Hennings A [47] studied the effect of physical activity on the pain areas of patients with multiple physical symptoms and found that short-term low-intensity exercise could increase PPT and reduce pain. Moreover, the level of physical activity is a related covariate. The stronger the level of physical activity is, the greater the change in the pain area. In the present research, high-intensity training could improve PPT more effectively in CLBP than low-intensity training. In a prospective study of Gupta A [48], a decrease in PPT and an increase in tender points were found to be the predictors of chronic generalized pain. Low PPT may be the result of pain and related factors. McPhee ME [49] found that as the duration of pain experienced by patients with LBP increased, their PPT decreased, and the residual pain caused by external stimulation lasted longer. The experiments in the present study showed that transverse abdominal muscle training could reduce the radiation range of waist pain and increase PPT, thus providing new evidence of the effectiveness of core muscle training in treating CLBP.

Some researchers [50] pointed out that the CLBP population may have different degrees of core muscle strength impairment. Targeted exercise training could improve the coordination and recruitment rate of the core muscles that support the spine, thereby improving the coordination and stability of the spine. Specialized TrA training for patients with CLBP could effectively increase the recruitment rate of TrA (7.8%), which is better than that of ordinary exercise (4.9%) and spinal manipulation therapy (3.7%). A significant medium correlation was also found between the improvement of the recruitment rate of TrA and the reduction in pain and dysfunction (r = − 0.35; 95% CI 0.02–0.62) [51]. Some scholars [52, 53] pointed out a delay in the feedforward activation of transversus abdominis in a population with CLBP. Targeted muscle training could improve feedforward postural strategies, and the magnitude of its effect depends on the type and quality of sports training. The experiment in the present study confirmed the results of previous studies.

After TrA training, the blood circulation of the waist core muscles is accelerated, which could promote the absorption of pain inflammatory factors [54]. After the pain is reduced, it could promote improvement of waist proprioception [55]. Studies showed that even a small amount of exercise may cause changes in the cerebral cortex and increase motor evoked potentials [56]. Increased training may promote functional reorganization of the somatosensory cortex [57]. The plasticity of the brain facilitates exercise learning and improves daily lumbar and pelvic spine control without pain [58]. Changes in other peripheral substances such as beta-endorphin, may be one of the physiological mechanisms of TrA training analgesia. Studies have shown that medium to high-intensity core muscle training for patients with chronic pain may promote the release of endogenous opioids, cause endogenous analgesia and improve PPT [59, 60]. This phenomenon is a virtuous circle, and core muscle training is its beginning [61].

## Conclusion

Transversus abdominis training could increase the PPT of the waist core muscles of patients with CLBP and reduce the radiation range of pain. High-intensity training could activate more waist core muscles and has more benefits than low-intensity training. The most obvious improvement in body surface pain radiation was observed after high-intensity training. This result is of great importance to the formulation of exercise prescriptions for patients with CLBP, and it provides references for major guidelines in listing exercise as the main treatment for CLBP.

However, the sample size of this study is relatively small, and the age group of the subjects is relatively concentrated. Follow-up studies should consider further increasing the sample size to make the research results representative. In addition, long-term intervention study was not conducted, and the effect of long-term intervention on PPT is unclear. These problems could be addressed in the follow-up study.

## Data Availability

The datasets generated and analyzed in this study are not available because the entire research under these funds has not been openly published yet, and this paper is one of the subtopics. However, they are available from the corresponding author upon reasonable request.
